# Temporal Transcriptomic Responses of *Turbinaria peltata* to Repeated Marine Heatwaves and Recovery Cycles

**DOI:** 10.3390/biology15211929

**Published:** 2026-10-24

**Authors:** Yanping Zhang, Yulu Sun, Xin Zhai, Jie Zhou, Li Liu

**Affiliations:** 1 College of Fisheries, Guangdong Ocean University, Zhanjiang 524088, China; ypzhang@gdou.edu.cn (Y.Z.); zhoujielyg@sina.com (J.Z.); 2 South China Sea Research Center for the Conservation of Rare and Endangered Marine Life, Guangdong Ocean University, Zhanjiang 524088, China; 3 Key Laboratory of Aquaculture in the South China Sea for Aquatic Economic Animal of Guangdong Higher Education Institutes, College of Fishery, Guangdong Ocean University, Zhanjiang 524088, China

**Keywords:** coral, marine heatwaves, transcriptome, thermal acclimatisation, oxidative stress, *Turbinaria peltata*

## Abstract

Marine heatwaves (MHWs) are escalating threats to coral reef ecosystems, yet the host transcriptomic mechanisms underlying acclimatization to repeated thermal stress remain poorly understood. Using de novo RNA sequencing, we tracked the temporal transcriptomic responses of the scleractinian coral *Turbinaria peltata* across two consecutive MHW-recovery cycles. The second heatwave elicited fewer differentially expressed genes (DEGs) than the first (279 vs. 379), whereas the second recovery phase showed the largest DEG set (399). Functionally, the first heatwave triggered acute stress defense and signal suppression, while the first recovery engaged extracellular matrix remodeling and calcium-mediated tissue repair, indicating incomplete restoration. The largest DEG set during the second recovery indicates that recurrent thermal stress imposes cumulative physiological costs demanding more extensive molecular reorganization—spanning metabolic reprogramming, mTOR and Wnt signaling, and sustained oxidative-stress management. Together with prior physiological and microbiome evidence in this species, our results support a model in which repeated heat exposure progressively shifts the coral host from acute shock defense responses toward compensatory metabolism and structural repair. Recovery thus emerges as an active but increasingly costly molecular adjustment under recurrent MHW regimes. These findings identify candidate molecular markers for evaluating coral resilience under intensifying MHW regimes.

## Simple Summary

Coral reefs are suffering from increasingly frequent marine heatwaves caused by climate change. While many studies have examined how corals respond during heat stress, less is known about how they recover afterwards, especially when heatwaves occur repeatedly. In this study, we exposed the reef-building coral *Turbinaria peltata* to two consecutive heatwave–recovery cycles and tracked changes in its gene expression. We found that after the first heatwave, corals initiated repair but did not fully recover. Surprisingly, after the second heatwave, even though the heat stress caused less physiological damage than the first, the corals required even greater gene expression activity to recover. This suggests that repeated heatwaves impose a cumulative legacy effect, where the cost of recovery accumulates over time. Our findings highlight that recovery is not a passive return to normal but an active, energy-consuming process—and that repeated stress can gradually erode a coral’s ability to bounce back.

## 1. Introduction

Coral reefs support exceptionally high biodiversity and provide critical ecosystem services, including coastal protection, fisheries support, and tourism, with an estimated global value exceeding $30 billion annually [1,2,3]. However, global climate change is fundamentally altering the physical and biological conditions these ecosystems require. Marine heatwaves (MHWs), defined as temperatures exceeding the 90th percentile of a 30-year climatological baseline for five or more days [4], have become a major threat to coral reefs, driving widespread bleaching and accelerating reef degradation worldwide [5]. Recent analyses indicate a global increase in the frequency and intensity of MHWs across coral reef regions [6], and climate projections suggest that these events will continue to intensify [7]. Notably, many corals are now exposed to successive heatwaves at increasingly short intervals [8,9], yet how the host transcriptome dynamically responds across repeated MHW-recovery cycles, and whether recovery between events is complete or progressively compromised, are questions that remain poorly understood.

At the molecular level, acute thermal stress triggers well-documented transcriptomic responses, including up-regulation of *heat shock proteins* (e.g., *HSP70*, *HSP20*), genes encoding antioxidant enzymes such as *superoxide dismutase* (*SOD*), *catalase*, and *glutathione S-transferase* (*GST*), as well as immune- and apoptosis-related gene networks [10,11,12]. Studies further reveal disruption of calcium homeostasis, suppression of intercellular signaling pathways, and down-regulation of developmental genes during initial thermal exposure [13,14]. While these findings substantially advance our understanding of single-event bleaching, they provide limited insight into how transcriptional programs are modulated across successive heat-recovery cycles.

Survivors of earlier bleaching events often exhibit reduced severity during later heatwaves, indicating thermal acclimatization [15,16]. On the Great Barrier Reef, back-to-back marine heatwaves promoted higher survival and thermal thresholds, albeit with metabolic trade-offs including shifts from autotrophy toward greater heterotrophy [17]. At the transcriptomic level, rapid acclimation is accompanied by extensive gene-expression remodeling, the magnitude and direction of which depend on prior thermal history [18]. Recent multi-omics work in *Pocillopora damicornis* further demonstrated stage-specific transcriptome shifts and concurrent DNA methylome reprogramming [19]. Repeated heat challenge further elicits a qualitatively distinct regulatory state—characterized by altered profiles of classical stress markers and chromatin-associated genes—rather than simply attenuation of the initial response [20]. Nevertheless, most molecular investigations have focused on the heat-stress phase itself, leaving the transcriptional dynamics of recovery, and how recovery periods contribute to acclimatization, largely unresolved.

Elucidating these recovery-phase dynamics is especially important for marginal and high-latitude reef systems, historically regarded as thermal refugia but now experiencing more frequent and intense marine heatwaves [21,22]. The 2020 mass bleaching event in the northern South China Sea demonstrated this vulnerability: over 81% of surveyed stations recorded bleaching, with an average prevalence of 51.14% [23], and cumulative thermal stress reached approximately 20 °C-weeks, substantially impairing the thermal refugia potential of these marginal reefs [22]. *Turbinaria peltata*, a dominant reef-building coral in the Xuwen Coral Reef National Nature Reserve of the northern South China Sea, provides an excellent model system for examining coral resilience to repeated thermal stress in such marginal environments.

We have previously demonstrated that *T. peltata* undergoes measurable physiological and microbiome changes under two successive heatwaves separated by recovery periods, including attenuated antioxidant enzyme responses during the second heatwave and a progressive shift from potentially pathogenic to more beneficial bacterial consortia [24]. Although these organism- and community-level patterns suggest acclimatization, they do not reveal the host transcriptional programs that regulate and coordinate these adjustments. To bridge this gap, the present study employs de novo RNA sequencing (RNA-seq) to (i) identify differentially expressed genes and enriched pathways underpinning coral responses to recurrent heat stress, and (ii) characterize temporal gene expression dynamics across initial heat exposure, recovery, and subsequent heat challenge. These analyses aim to advance our understanding of the molecular mechanisms that govern coral resilience to recurring marine heatwaves.

## 2. Materials and Methods

### 2.1. Sample Collection

*T. peltata* specimens were collected from the Xuwen Coral Reef National Nature Reserve (109°55′26 E, 20°16′00 N). The corals were temporarily housed in a 200 L tank with the following water conditions: temperature of 26 °C, pH of 8.0, and salinity of 33 PSU for a duration of 7 days. Artificial seawater was prepared using coral salt from Aquarium Systems in France. The corals were illuminated using a full-spectrum LED lamp (Maxspect, Guangzhou, China) with a light–dark ratio of 12 h:12 h and a PAR intensity of 200 µmol (m^−2^ s^−1^). After the acclimation, *T. peltata* colonies were fragmented into 24 pieces and placed in another 200 L tank for an additional week until the corals extended tentacles.

### 2.2. Experimental Design

**Figure 1:**
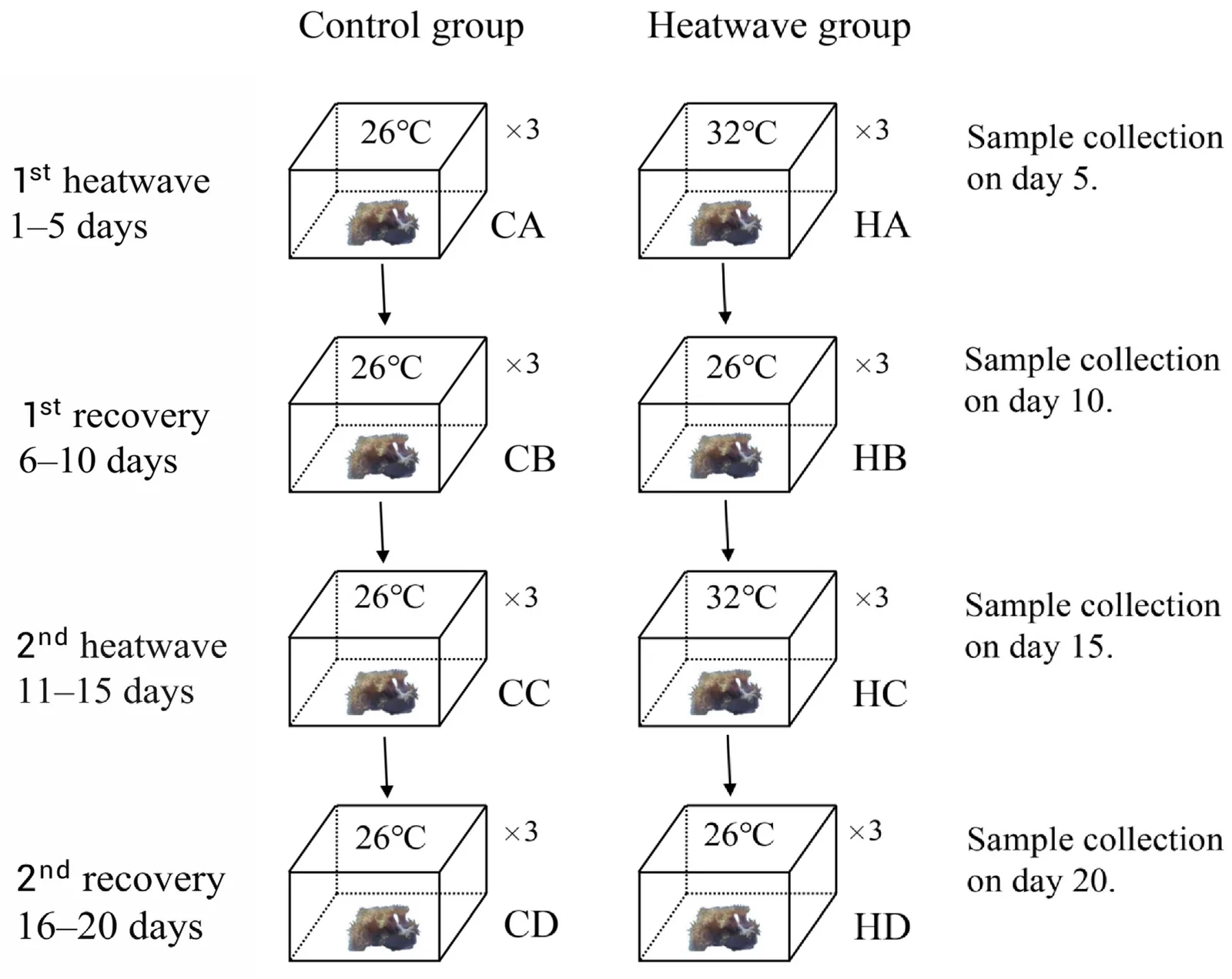
Design of the culture experiment. Coral nubbins were divided into control (“C”) and heatwave (“H”) groups and exposed to two consecutive MHW-recovery cycles. Abbreviations: the first letter denotes the treatment group (C, control; H, heatwave) and the second letter denotes the sampling stage (A, day 5; B, day 10; C, day 15; D, day 20).

To simulate the impact of heatwaves on corals, a heatwave treatment was conducted by manipulating the seawater temperature over a specific period (Figure 1). Coral nubbins were randomly divided into 6 aquariums (4 nubbins per aquarium; 24 nubbins in total), each with a capacity of 20 L. Two experimental groups were established: the control group (“C”) and the heatwave group (“H”), with 3 aquariums per group (12 nubbins per group; *n* = 3 aquariums per group), based on historical high-temperature extremes recorded in Xuwen over the past 10 years (source: http://gd.cma.gov.cn), and the ramp from 26 to 32 °C over 5 days (~1.2 °C day^−1^) is comparable to heating rates used in other experimental studies simulating MHW-like thermal stress in the northern South China Sea [24].

From day 1 to day 5, a microcomputer digital thermostat (XH-W1308, Tyrell, Shenzhen, China) was used to gradually increase the seawater temperature from 26 °C to 32 °C, representing the first heatwave. Subsequently, from day 6 to day 10, the seawater temperature was reduced back to 26 °C, simulating the first recovery period. Starting from day 11 to day 15, the seawater temperature was increased again to 32 °C to model a second heatwave. Finally, from day 16 to day 20, the seawater temperature was lowered to 26 °C to simulate the second recovery period. In the control treatment, the seawater temperature was maintained constantly at 26 °C throughout the entire exposure period.

Specific dates were assigned as “day 5” (end of the first heatwave), “day 10” (end of the first recovery period), “day 15” (end of the second heatwave), and “day 20” (end of the second recovery period), and were denoted as “A”, “B”, “C”, and “D”, respectively. From each group, three corals were randomly selected for sampling.

### 2.3. Transcriptome Sequencing and Analysis

Following the manufacturer’s protocol, coral tissues were ground using scissors and mortar with liquid nitrogen. Total RNA was extracted from the homogenized samples using TRIzol reagent (CW0580S, CWBIO, Taizhou, China). RNA concentration was quantified using a Nanodrop 2000 spectrophotometer (Thermo Fisher Scientific, Waltham, MA, USA), and integrity was assessed with an Agilent 2100 Bioanalyzer (Agilent Technologies, Santa Clara, CA, USA) using the LabChip GX assay (Revvity, Waltham, MA, USA). RNA samples meeting quality thresholds were subsequently sent to Beijing Biomarker Technologies Corporation (Beijing, China) for de novo transcriptome sequencing. Libraries were quantified using a Qubit 3.0 fluorometer (Thermo Fisher Scientific, Waltham, MA, USA) (≥1 ng/μL), and insert sizes were examined with a Qsep400 (BiOptic Inc., Changzhou, China) high-throughput analysis system. Library concentration was then accurately quantified by Q-PCR (>2 nM). Sequencing was performed on the Illumina NovaSeq 6000 platform (Illumina, San Diego, CA, USA) in PE150 mode. Raw reads were filtered to remove (1) reads containing adapters, (2) reads with more than 10% ambiguous bases (N), and (3) reads with more than 50% of bases having a quality score ≤ 10. Clean reads were retained in FASTQ format for downstream analysis. De novo assembly was performed using Trinity (v2.5.1) [25] with the following parameters: --min_contig_length 200 and --group_pairs_distance 500; all other parameters were set to default. For isoforms of the same gene, the longest transcript was selected as the representative unigene. Identical sequences were removed using CD-HIT (v4.8.1) [26], with parameters: -c 0.9 -n 10, after which the remaining sequences were clustered using TGICL, with sequences showing greater than 90% similarity and greater than 35 bp overlap merged. Unigenes were aligned against the NR [27], Swiss-Prot [28], COG [29], KOG [30], eggNOG [31] and KEGG databases [32] using DIAMOND (v2.0.4) [33], with the following parameters: -k100-e value 1e-5-f5. KEGG pathway assignments were then obtained by mapping these alignments to KEGG Orthology terms using KOBAS [34]. GO annotations were derived using InterProScan (5.34–73.0) [35], with the following parameters: -appl Pfam -goterms -iprlookup -pa -f xml -dp-, against the InterPro database. Predicted amino acid sequences were aligned against the Pfam database using HMMER (v3.1b2) [36], with the following parameters: E-value ≤ 1 × 10^−10^. Gene expression levels were quantified based on read counts. Differential expression analysis was performed using DESeq2 (v1.30.1) [37], with the following parameters: default: test = “Wald”, fit Type = “parametric”, including normalization of all genes across libraries and identification of differentially expressed genes (DEGs). DEGs were defined as those with |log2FC| ≥ 1 and *p* < 0.01. GO and KEGG enrichment analyses were performed on the identified DEGs using TBtools (v2.303) [38] based on the hypergeometric test. *p* values were adjusted for multiple testing using the Benjamini–Hochberg method, and terms or pathways with adjusted *p* < 0.05 were considered significantly enriched.

To characterize the transcriptional dynamics across the four experimental stages (HA-HB-HC-HD), co-expression analysis was performed using the co-expression module on the BMKCloud platform (www.biocloud.net). Expression values were averaged across the three biological replicates for each stage before clustering. Genes with similar expression patterns were grouped based on Euclidean distance using K-means clustering. The optimal number of clusters was determined using the NbClust package (min.nc = 2, max.nc = 20, distance = “euclidean”, method = “complete”, index = “kl”), and the resulting k was used for K-means clustering.

The sequencing data generated in this study have been deposited in the NCBI database under BioProject accession number PRJNA1050673.

### 2.4. Reverse Transcription Quantitative PCR (qPCR)

To validate the reliability of transcriptomic data, eight differentially expressed genes representing the main functional categories identified from the sequencing analysis were selected for RT-qPCR verification. Using the same RNA batches as those employed for transcriptome sequencing, cDNA was synthesized through reverse transcription. The diluted cDNA samples were analyzed in triplicate, with *β-actin* as the reference gene. Primers were designed using Primer 6 (Table 1). The thermal cycling protocol consisted of initial denaturation at 95 °C for 3 min; followed by 40 cycles of denaturation at 95 °C for 5 s, annealing at 58 °C for 15 s, and extension at 72 °C for 30 s (with fluorescence acquisition); with a final extension at 72 °C for 2 min. The specificity of PCR amplification was confirmed by melt curve analysis. Gene expression levels were calculated using the 2^−(ΔΔCt)^ method and presented as mean ± standard deviation [39].

## 3. Results

### 3.1. Transcriptome Sequencing Quality and Functional Annotation

**Figure 2:**
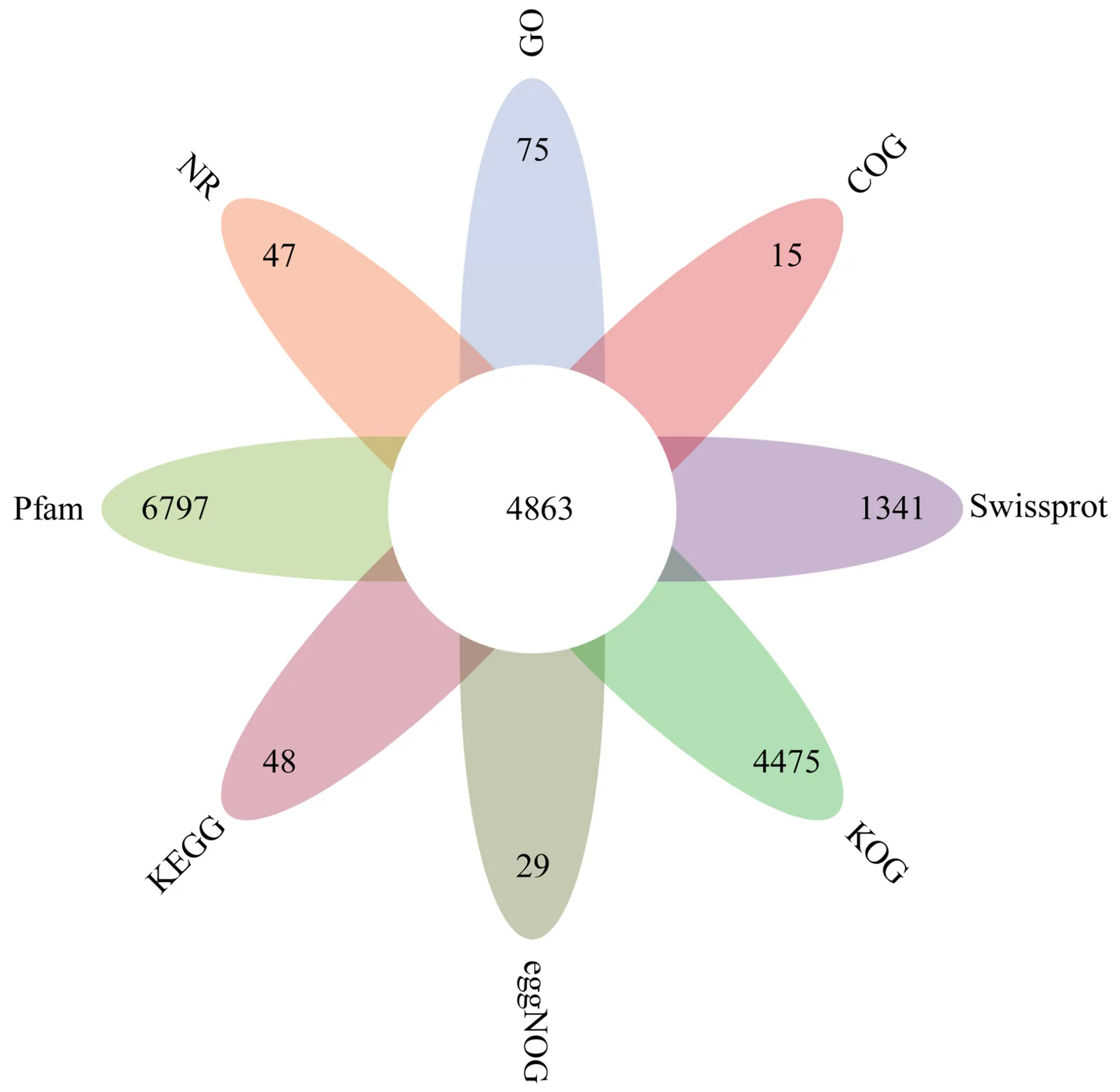
Venn diagram of Unigene annotation results. Numbers represent the number of genes annotated by a single database or by all databases together. A complete list of gene IDs and annotations for all expressed genes is provided in Supplementary Table S2. Abbreviations: Pfam: Protein Families Database; NR: Non-Redundant Protein Database; Swiss-Prot: UniProtKB/Swiss-Prot; GO: Gene Ontology; COG: Clusters of Orthologous Groups of proteins; KOG: Eukaryotic Orthologous Groups; eggNOG: evolutionary genealogy of genes: Non-supervised Orthologous Groups; KEGG: Kyoto Encyclopedia of Genes and Genomes.

RNA-seq analysis of 24 transcriptome samples generated a total of 153.32 Gb of high-quality clean data. After quality control filtering, 512,202,173 clean reads were retained, with a Q30 base percentage exceeding 93.16% and a GC content of 47.78% (Supplementary Table S1). De novo assembly was performed using Trinity, resulting in 99,972 unigenes. Among these, 7671 unigenes were longer than 1000 bp, and the N50 length was 1029 bp (Table 2). Functional annotation of the assembled unigenes was conducted against multiple public databases, including Swiss-Prot, COG, KOG, Gene Ontology (GO), Kyoto Encyclopedia of Genes and Genomes (KEGG), Pfam, and eggNOG. In total, 11,833, 7953, 14,573, 24,619, 19,370, 22,308, and 19,111 unigenes were successfully annotated in these databases, respectively, as shown in Figure 2.

### 3.2. Expression Trends of Shared Genes Across Recurrent Thermal Stress and Recovery

**Figure 3:**
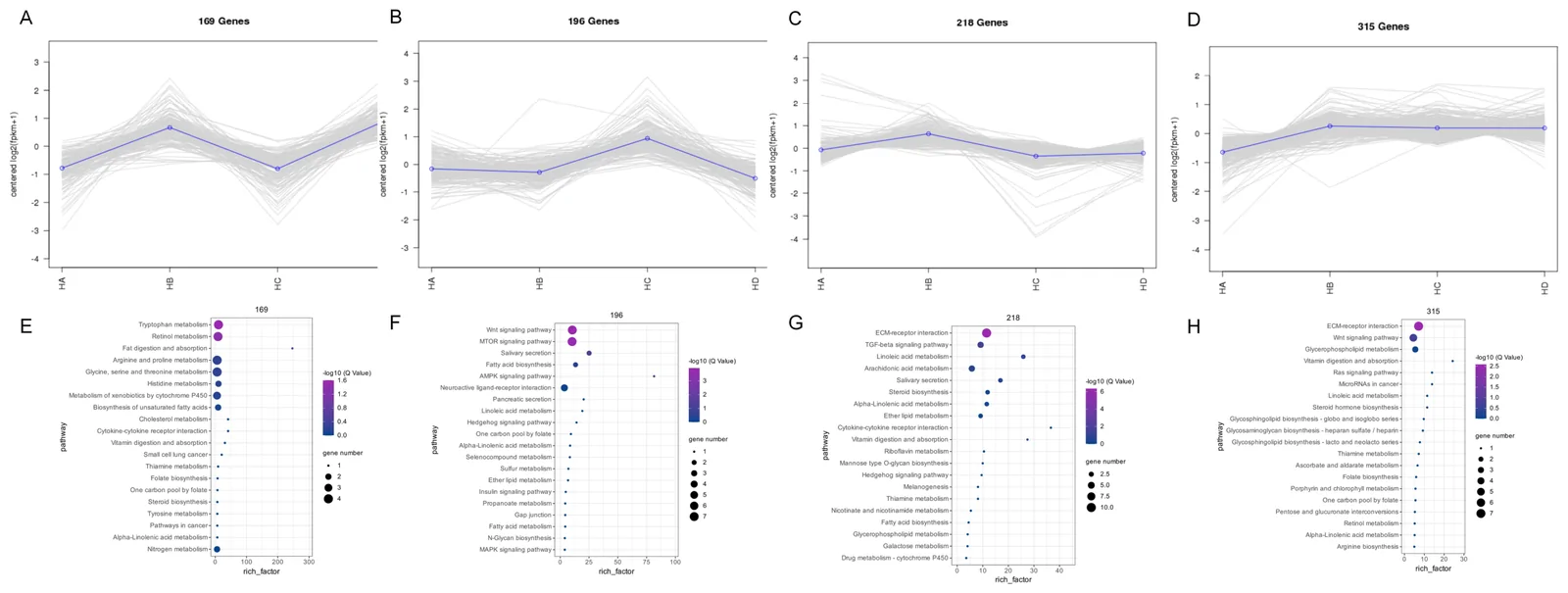
Co-expression trend analysis of genes across the four experimental stages and KEGG enrichment of genes within each trend. (**A**) Trend 1; (**B**) Trend 2; (**C**) Trend 3; (**D**) Trend 4; (**E**) KEGG enrichment of genes within trend 1; (**F**) KEGG enrichment of genes within trend 2; (**G**) KEGG enrichment of genes within trend 3; (**H**) KEGG enrichment of genes within trend 4. Genes were clustered by expression pattern using K-means clustering, yielding four trends. KEGG enrichment was performed on genes within each trend, and the top 20 pathways ranked by *p* value are shown for each trend. The detailed lists of genes in each trend are provided in Supplementary Table S3. Abbreviations: CA, *control group A* (day 5); HA, *heat stress group A* (day 5); CB, *control group B* (day 10); HB, *heat stress group B* (day 10); CC, *control group C* (day 15); HC, *heat stress group C* (day 15); CD, *control group D* (day 20); HD, *heat stress group D* (day 20).

To characterize the transcriptional dynamics of *T. peltata* across the four experimental stages, commonly expressed genes were clustered by expression trend. Four distinct trends were identified (Figure 3A–D). Trend 1 (169 genes) showed higher expression during both recovery phases and lower expression during both heatwave periods. Trend 2 (196 genes) peaked specifically during the second heatwave and remained low at all other stages. Trend 3 (218 genes) showed relatively stable expression across all four stages. Trend 4 (315 genes) was up-regulated from the first recovery phase and sustained through the second recovery.

KEGG enrichment analysis of genes within each trend revealed distinct functional associations (Figure 3E–H), with pathways considered significantly enriched at q < 0.05. Trend 1 was enriched in “tryptophan metabolism” and “retinol metabolism”; Trend 2 in “Wnt signaling pathway” and “mTOR signaling pathway”; Trend 3 in “ECM-receptor interaction” and “TGF-beta signaling pathway”; and Trend 4 in “ECM-receptor interaction”.

### 3.3. Differentially Expressed Genes (DEGs) in Transcriptome Sequencing

**Figure 4:**
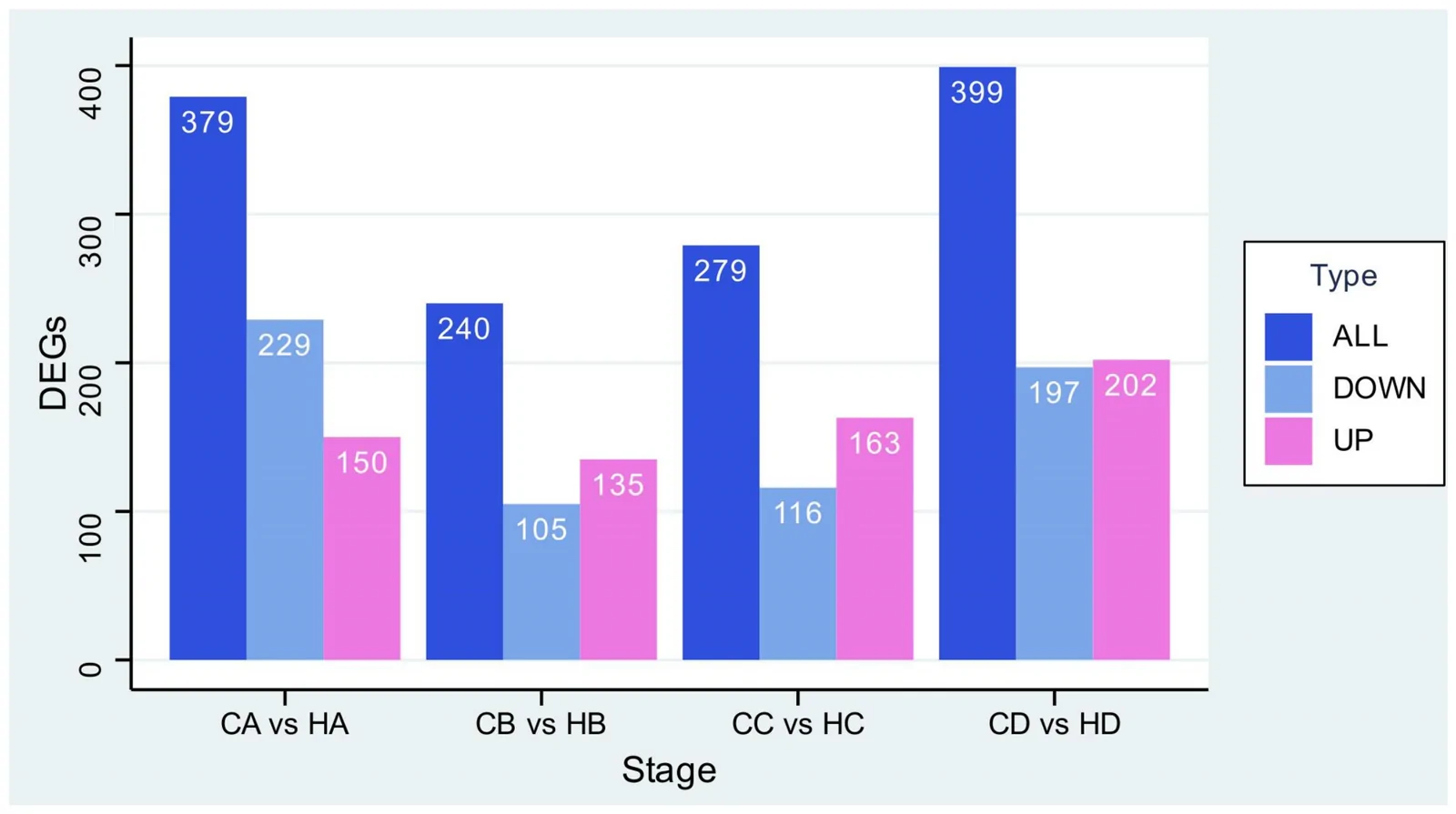
Statistics of differentially expressed genes (DEGs). Abbreviations: CA, *control group A* (day 5); HA, *heat stress group A* (day 5); CB, *control group B* (day 10); HB, *heat stress group B* (day 10); CC, *control group C* (day 15); HC, *heat stress group C* (day 15); CD, *control group D* (day 20); HD, *heat stress group D* (day 20).

Differentially expressed genes (DEGs) were identified based on a threshold of *p* < 0.01 and |Fold Change| ≥ 2. The temporal transcriptomic responses between the heatwave and control groups were subsequently examined across different experimental stages. At the end of the first heatwave exposure, a total of 379 DEGs were identified, including 229 down-regulated and 150 up-regulated genes. Following the subsequent recovery period, the number of DEGs decreased to 240, with 105 genes down-regulated and 135 up-regulated, indicating a partial transcriptional recovery. During the second heatwave exposure, 279 DEGs were detected, comprising 116 down-regulated and 163 up-regulated genes. Notably, after the second recovery period, the number of DEGs increased again to 399, with 197 down-regulated and 202 up-regulated genes, suggesting a more complex and intensified transcriptional response after repeated thermal stress, as shown in Figure 4.

### 3.4. GO Enrichment Analysis of Differentially Expressed Genes

**Figure 5:**
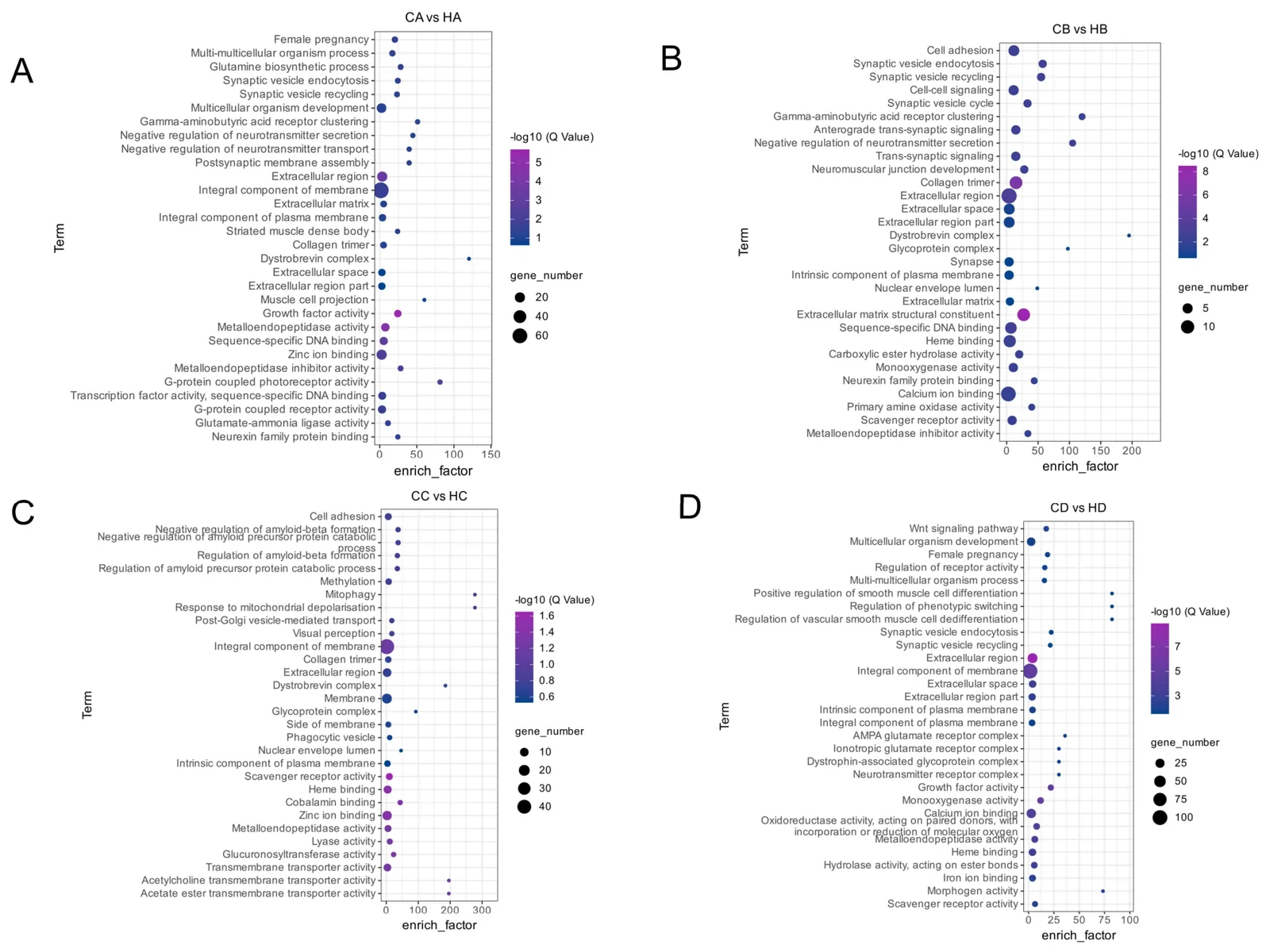
Top 10 enriched GO terms in the biological process (BP), cellular component (CC), and molecular function (MF) of differentially expressed genes for each experimental stage in *T. peltata*. (**A**) First heatwave (CA vs. HA); (**B**) first recovery (CB vs. HB); (**C**) second heatwave (CC vs. HC); (**D**) second recovery (CD vs. HD). The *x*-axis represents the enrichment factor, defined as the ratio of the proportion of differentially expressed genes (DEGs) annotated to a given GO term to the proportion of background genes annotated to the same term. The *y*-axis shows the enriched GO terms. The dot size corresponds to the number of genes, and the color indicates the enrichment significance (−log10 (*p*-value)). The detailed lists of GO enrichment of differentially expressed genes (DEGs) are provided in Supplementary Table S4. Abbreviations: the first letter denotes the treatment group (C, control; H, heatwave) and the second letter denotes the sampling stage (A, day 5; B, day 10; C, day 15; D, day 20).

At the end of the first heatwave exposure, a total of 15 Gene Ontology (GO) terms were significantly enriched (q < 0.05), comprising 3 biological process (BP), 2 cellular component (CC) and 10 molecular function (MF), with BP terms including “glutamine biosynthetic process”, MF terms including “growth factor activity”, “metalloendopeptidase activity” and “sequence-specific DNA binding” (Figure 5A). The majority of these enriched GO terms were associated with down-regulated genes. Although the first recovery stage had the fewest DEGs, it yielded the largest number of significantly enriched GO terms (171, q < 0.05), comprising 151 BP, 2 CC, and 18 MF terms, with BP terms including “cell adhesion”, “synaptic vesicle endocytosis”, “synaptic vesicle recycling”, and “cell-cell signaling” (Figure 5B). This apparent discrepancy was partly attributable to annotation redundancy: two unigenes (TRINITY_DN2944_c0_g2 and TRINITY_DN3403_c0_g1) together accounted for more than 70 of these BP terms. In contrast, after the second heatwave exposure, only 4 GO terms within MF were significantly enriched: “scavenger receptor activity”, “heme binding”, “cobalamin binding”, and “zinc ion binding” (q < 0.05) (Figure 5C), indicating a reduced but more focused functional response under repeated thermal stress. During the second recovery period, 70 GO terms were significantly enriched (q < 0.05), comprising 27 BP, 12 CC, and 31 MF terms, with MF terms including “growth factor activity”, “monooxygenase activity”, and “calcium ion binding” (Figure 5D). Notably, both recovery periods yielded more enriched GO terms than the heatwave phases, and the second recovery showed a more balanced distribution across BP, CC, and MF. Overall, heatwave exposure was associated with focused functional responses, whereas recovery—particularly the second recovery—showed broader functional engagement.

### 3.5. KEGG Pathway Enrichment Analysis of Differentially Expressed Genes

**Figure 6:**
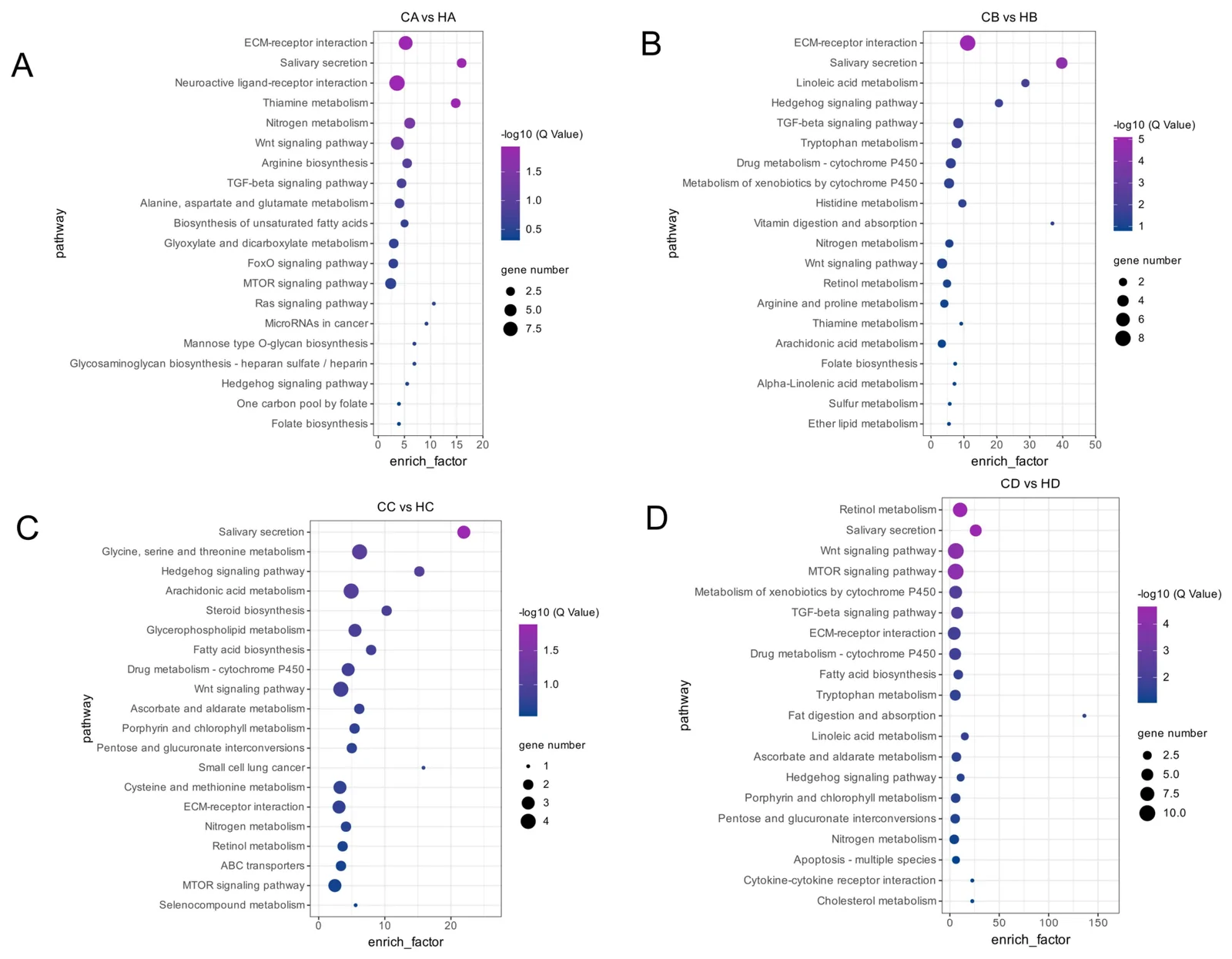
Top 20 enriched KEGG pathways of differentially expressed genes for each experimental stage in *T. peltata*. (**A**) First heatwave (CA vs. HA); (**B**) first recovery (CB vs. HB); (**C**) second heatwave (CC vs. HC); (**D**) second recovery (CD vs. HD). The *x*-axis represents the enrichment factor, defined as the ratio of DEGs annotated to a specific KEGG term to the total number of DEGs. The *y*-axis shows the enriched pathway terms. The dot size corresponds to the number of genes, and the color indicates the enrichment significance (−log10 (*p*-value)). Abbreviations: the first letter denotes the treatment group (C, control; H, heatwave) and the second letter denotes the sampling stage (A, day 5; B, day 10; C, day 15; D, day 20). The detailed lists of KEGG pathway annotations of differentially expressed genes are provided in Supplementary Table S5.

Kyoto Encyclopedia of Genes and Genomes (KEGG) pathway enrichment analysis revealed that differentially expressed genes (DEGs) were predominantly associated with “Environmental Information Processing” and “Metabolism”-related pathways under repeated heatwave exposure (Figure 6). At the end of the initial heatwave exposure, a total of 6 pathways were significantly enriched (q < 0.05), including “ECM–receptor interaction”, “Salivary secretion”, “Neuroactive ligand–receptor interaction” and “Thiamine metabolism”. Notably, most genes in these pathways were down-regulated, suggesting an overall suppression of intercellular signaling and extracellular communication under acute thermal stress (Figure 6A). In the first recovery period, 9 pathways were significantly enriched (q < 0.05), and DEGs were enriched in “ECM–receptor interaction”, “Salivary secretion” and “Linoleic acid metabolism”. In contrast to the heatwave phase, most genes in these pathways were up-regulated, indicating active physiological restoration and metabolic reactivation during recovery (Figure 6B). During the second heatwave exposure, only 1 pathway, “Salivary secretion”, was significantly enriched (q < 0.05), with most genes in this pathway being up-regulated (Figure 6C). In the second recovery period, 16 pathways were significantly enriched (q < 0.05); DEGs were significantly enriched in “Retinol metabolism”, “Salivary secretion”, “Wnt signaling pathway” and “mTOR signaling pathway”, as shown in Figure 6D. Notably, except for the first heatwave exposure, most metabolism-associated pathways were enriched with up-regulated genes, suggesting enhanced energy allocation and regulatory rebuilding during recovery from repeated stress exposure.

**Figure 7:**
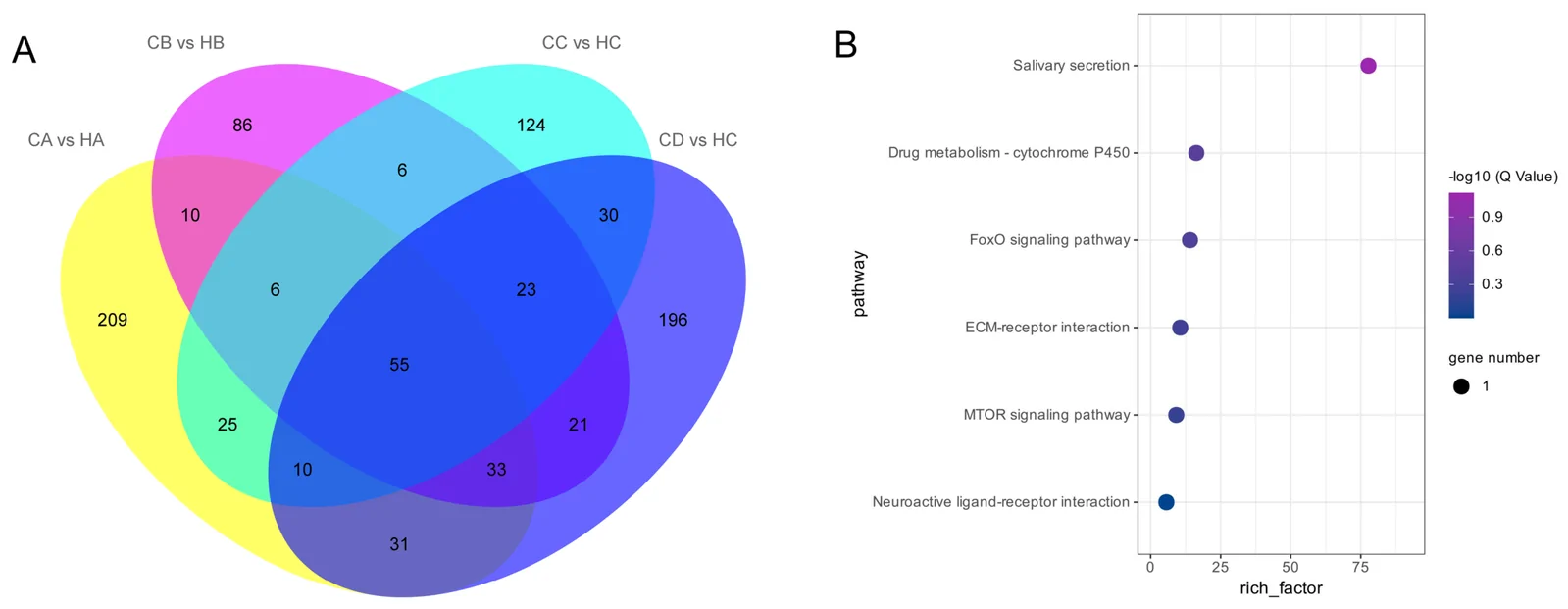
Venn diagram of DEGs identified across the four experimental stages and KEGG enrichment of the shared 55-gene set. (**A**) Venn diagram showing DEGs shared among and unique to the four stages. The numbers indicate the count of DEGs shared among or unique to the different stages. (**B**) KEGG enrichment analysis of the 55 genes shared across all four stages. The *x*-axis represents the enrichment factor, defined as the ratio of unigenes annotated to a specific KEGG term to the total number of unigenes. The *y*-axis shows the enriched pathway terms. The dot size corresponds to the number of genes, and the color indicates the enrichment significance (−log10 (*p*-value)). Significantly enriched pathways were defined as *p* < 0.05. Abbreviations: the first letter denotes the treatment group (C, control; H, heatwave) and the second letter denotes the sampling stage (A, day 5; B, day 10; C, day 15; D, day 20).

When we performed Venn diagram analysis on the DEGs between coral samples at different treatment stages and the control group, a total of 55 genes were commonly differentially expressed in all four stages (Figure 7A), and KEGG enrichment of these shared DEGs identified the “salivary secretion pathway” ranked first in the enrichment results (Figure 7B). Further analysis of the DEGs annotated to this pathway revealed that 3 of the 6 genes exhibited a greater magnitude of change (log2FC) during the second heatwave than during the first, and 5 of the 6 genes showed a stronger response during the second recovery period than during the first recovery period (Supplementary Table S6). This pattern within the “salivary secretion pathway” mirrors the broader transcriptional trend observed across the four stages. Overall, these results indicate a dynamic shift from signal suppression under acute heat stress to metabolic reprogramming and pathway reactivation during recovery, particularly under repeated thermal exposure.

### 3.6. Analysis of Differentially Expressed Genes

**Figure 8:**
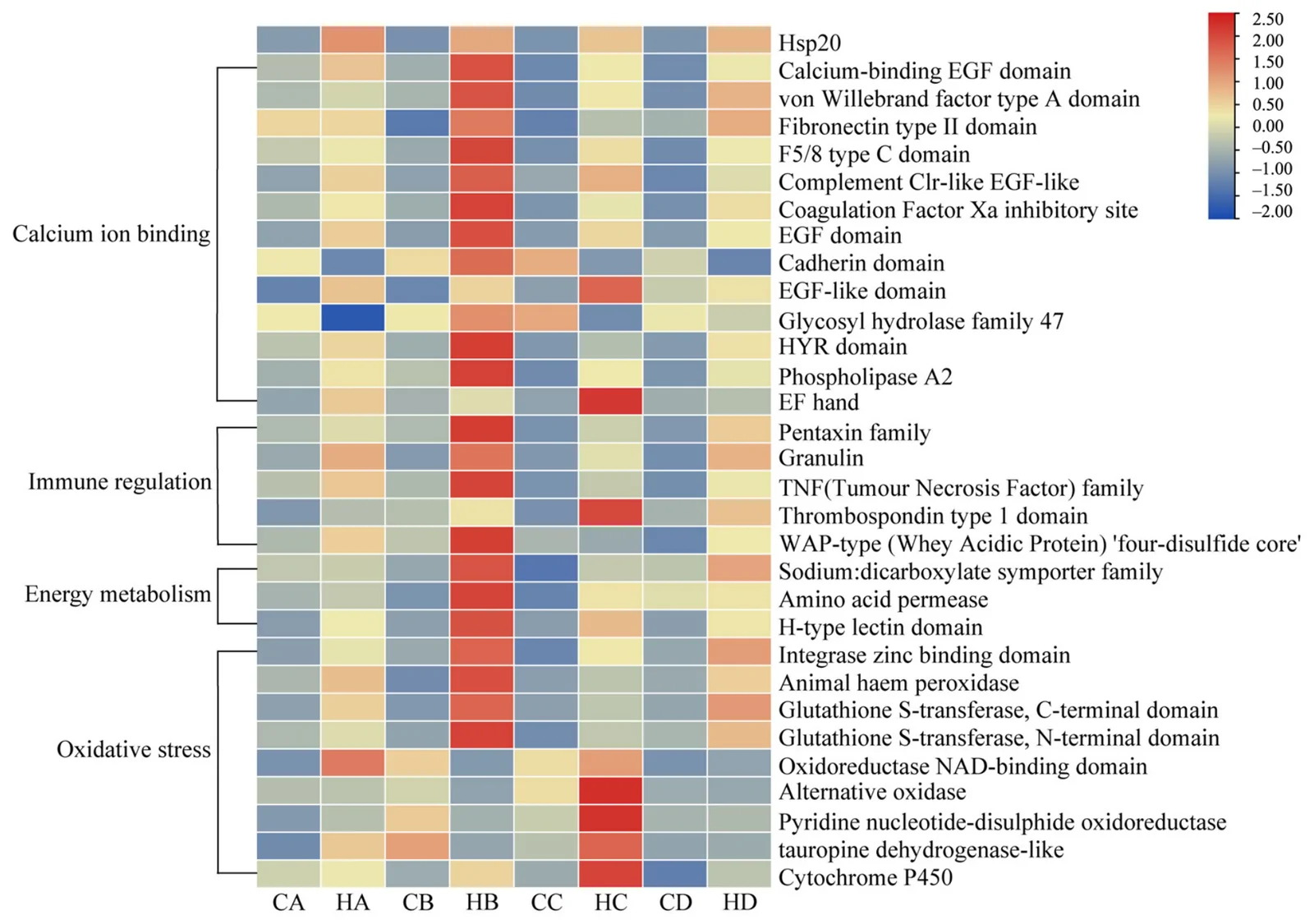
Differentially expressed gene expression profiles in *T. peltata*. Values are row-scaled Z-scores across the eight samples (CA, HA, CB, HB, CC, HC, CD, HD). Abbreviations: CA, *control group A* (day 5); HA, *heat stress group A* (day 5); CB, *control group B* (day 10); HB, *heat stress group B* (day 10); CC, *control group C* (day 15); HC, *heat stress group C* (day 15); CD, *control group D* (day 20); HD, *heat stress group D* (day 20).

To further elucidate the effects of repeated marine heatwaves on *T*. *peltata*, 32 representative DEGs associated with immune regulation, energy metabolism, oxidative stress, and calcium-binding functions were selected for detailed analysis. The results showed persistent differential expression of *the heat shock protein Hsp20* across all stages of the heatwave treatments, indicating a sustained role in thermal stress response. Genes associated with calcium ion binding, immune regulation, nutrient regulation, and oxidative stress were predominantly up-regulated during the first recovery period, suggesting active physiological restoration and immune rebalancing following the initial heat stress. In contrast, genes containing *EGF-like domains*, *EF-hand motifs*, *thrombospondin type 1 domains*, as well as those encoding *alternative oxidase*, *pyridine nucleotide-disulphide oxidoreductase*, *tauropine dehydrogenase-like proteins*, and *cytochrome P450* were mainly up-regulated during the second heatwave exposure, suggesting an enhanced metabolic and stress-defense response under repeated thermal challenge. Notably, genes harboring *oxidoreductase NAD-binding domains* were significantly expressed during both heatwave events, highlighting their potential role in maintaining redox homeostasis under thermal stress (Figure 8). Collectively, these results suggest a shift from recovery-oriented regulation after the initial stress to a more metabolically reprogrammed response under repeated heatwave exposure.

### 3.7. Reverse Transcription Quantitative PCR

**Figure 9:**
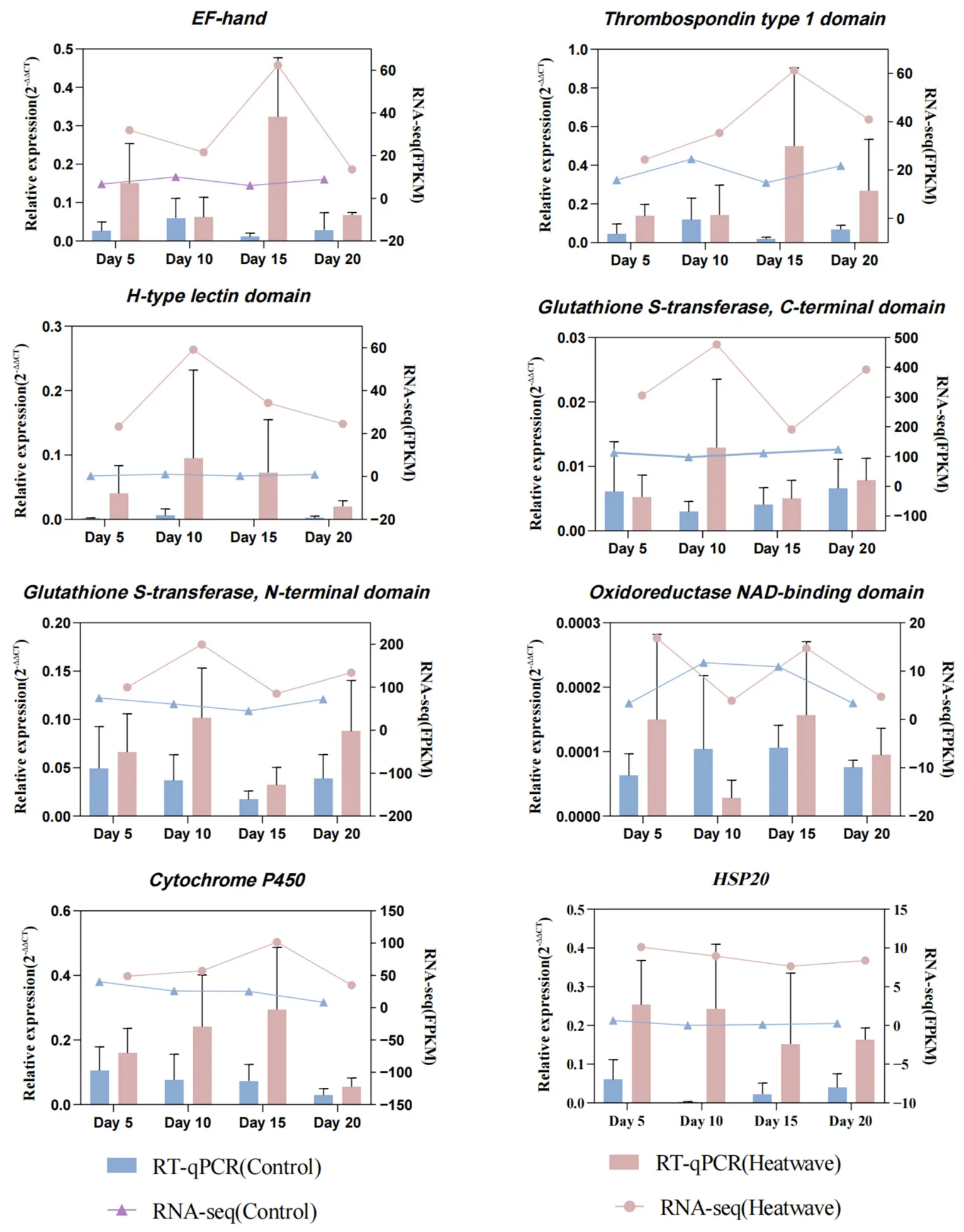
RT-qPCR validation results. Eight differentially expressed genes (*EF-hand*, *Thrombospondin type 1 domain*, *H-type lectin domain*, *Glutathione S-transferase C-terminal domain*, *Glutathione S-transferase N-terminal domain*, *Oxidoreductase NAD-binding domain*, *Cytochrome P450*, and *HSP20*) were validated by RT-qPCR across four sampling stages (day 5, 10, 15, and 20). Bars represent RT-qPCR expression values (mean ± SE, *n* = 3 biological replicates) for the control (blue) and heatwave (pink) groups, and lines represent the corresponding RNA-seq expression trends. *β-actin* was used as the reference gene for RT-qPCR.

To validate the transcriptome sequencing data, eight differentially expressed genes were randomly selected from the sequencing dataset for Reverse transcription quantitative PCR (RT-qPCR) verification. Each gene was analyzed with three biological replicates, and the results were compared between transcriptomic sequencing and RT-qPCR. As shown in Figure 9, the expression trends of *EF-hand*, *H-type lectin domain*, *Thrombospondin type 1 domain*, *Glutathione S-transferase*, *C-terminal domain*, *Glutathione S-transferase*, *N-terminal domain*, *Oxidoreductase NAD-binding domain*, *Cytochrome P450*, and *HSP20* obtained by RT-qPCR were generally consistent with the transcriptome sequencing data, supporting the reliability of the sequencing results.

## 4. Discussion

Marine heatwaves (MHWs) are becoming increasingly frequent, intense, and prolonged under global climate change, posing a major threat to reef-building corals through repeated bleaching events and ecosystem degradation [9,40]. However, coral responses to recurrent thermal stress are not simply determined by stress intensity, but are shaped by thermal history, physiological condition, and the capacity for molecular acclimatization [17]. In our previous study, we demonstrated that *T. peltata* exhibited physiological adjustment and microbiome restructuring following repeated MHW exposure, including reduced oxidative responses during the second heatwave and a shift toward more beneficial microbial communities during recovery [24]. Here, we extend these observations by characterizing host transcriptional profiles across two consecutive heat stress events and intervening recovery periods.

Across the four experimental stages, the second heatwave elicited fewer DEGs than the first, while trend analysis revealed a response involving newly induced, persistently activated, and repeatedly engaged gene sets. Together, these patterns suggest a reorganized rather than simply attenuated transcriptional response, consistent with the concept of ecological memory [8] and distinct from the classical priming model [41]. Recovery appears to play an active role in this process, rather than representing a passive return to baseline.

### 4.1. Transcriptomic Changes in T. peltata During Recurrent Thermal Stress

The first heatwave induced an acute transcriptional response in thermally inexperienced *T. peltata*, with 379 DEGs reflecting broad cellular perturbation and activation of stress defense mechanisms. Functional enrichment analysis revealed suppression of signaling-related pathways, including “growth factor activity” and “Wnt signaling”, suggesting that acute thermal stress redirected cellular resources from growth and physiological maintenance toward survival and damage control. Meanwhile, the persistent expression of molecular chaperones such as *Hsp20* indicates enhanced proteostasis mechanisms to mitigate heat-induced protein damage. Similar genome-wide transcriptional alterations have been reported in other scleractinian corals exposed to rapid thermal elevation, characterized by activation of molecular chaperones and suppression of normal cellular processes [18]. Consistent with our previous study [24], the first heatwave caused the most severe physiological impairment, including a 79.44% reduction in zooxanthellae density, a 33.67% decrease in chlorophyll a content, and significant increases in GST, SOD, CAT, and caspase-3 activities. This concordance between the magnitude of transcriptional change and physiological disruption supports the interpretation that the 379 DEGs during the first heatwave reflected a genuine acute stress response.

The second heatwave resulted in 279 DEGs, a value intermediate between the first recovery and initial heatwave phases. Importantly, this reduction in DEG number should not be interpreted as evidence of reduced stress severity, because DEG abundance alone does not directly reflect the magnitude of physiological disturbance. Instead, the altered transcriptional profile likely represents a reprogrammed regulatory state shaped by previous thermal exposure. Supporting this interpretation, the second heatwave caused substantially less physiological damage than the initial event. Zooxanthellae density decreased by only 69.71% compared with 79.44% during the first heatwave, chlorophyll a declined by 17.63% compared with 33.67%, and GST and caspase-3 responses were significantly attenuated [24]. These patterns support a reorganized transcriptional response, in which previous thermal exposure alters the mode of the subsequent response rather than simply enhancing overall tolerance. Consistent with this view, thermal acclimation in *Pocillopora damicornis* was associated with stage-specific transcriptomic remodeling and DNA methylome changes, suggesting that prior thermal experiences may be partially retained through epigenetic regulation [19]. Similarly, Caribbean corals exposed to thermal preconditioning exhibited fewer transcriptional changes during subsequent heat stress, accompanied by reduced expression of oxidative stress and immune-related markers and enhanced regulation of histone modification pathways [20]. Together, these findings indicate that prior thermal exposure does not simply suppress coral stress responses, but instead reshapes transcriptional regulation. This reorganized regulatory state was evident in the functional enrichment profile during the second heatwave, which was dominated by metabolic pathways, including arachidonic acid metabolism and glycine, serine, and threonine metabolism. This metabolic orientation suggests that *T. peltata* had transitioned from an acute stress-defense state toward compensatory metabolic adjustment. Similar metabolic flexibility has been observed in heat-tolerant corals, where surviving colonies adjust energy acquisition and utilization strategies following thermal disturbance [17]. Collectively, these host transcriptional adjustments were accompanied by holobiont-level changes: the concurrent enrichment of stress-tolerant bacterial taxa, including *Achromobacter arsenitoxydans* and *Halomonas desiderata*, during the second heatwave may have further contributed to host metabolic stability by supporting oxidative stress regulation and nutrient cycling [24].

### 4.2. Transcriptomic Changes in T. peltata Across the Recovery Process

The trend analysis also provides insight into the recovery process. The repeated engagement of recovery-associated genes across both recovery phases, together with the persistent activation of a large gene set from the first recovery through the second, suggests that recovery is not a passive return to baseline but an active, recurrent process [41,42]. Consistent with this, the second recovery phase showed the largest number of DEGs and was enriched in pathways related to tissue repair and metabolic reorganization.

Studies have found that the rate of return to baseline levels of gene expression following thermal stress is linked to thermal tolerance in coral [43,44,45,46]. Rapid transcriptomic recovery is beneficial when stress-induced changes in gene expression incur significant costs to life history traits. In our study, during the first recovery period, the number of DEGs decreased to 240, indicating partial transcriptional restoration. However, the persistence of over 200 DEGs suggested that recovery was incomplete rather than a full return to the pre-stress state. Previous studies have shown that *Ruditapes philippinarum* recovery following heat stress can remain constrained by prolonged energetic depletion, oxidative imbalance, and microbial dysbiosis even after thermal conditions normalize [47], which may explain the residual transcriptional activity observed here. Notably, the first recovery transcriptome was enriched in extracellular matrix (ECM) structural constituents, calcium-ion binding, ECM–receptor interaction, and cytochrome P450-mediated metabolism. These pathways indicate active tissue remodeling, biomineralization repair, and detoxification processes, suggesting that post-stress restoration is an energetically demanding process requiring substantial transcriptional investment, rather than a passive return to homeostasis. This interpretation agrees with our physiological and microbiome observations, where zooxanthellae density and chlorophyll a content remained below control levels and stress-associated microbial signatures persisted during first recovery [24].

Interestingly, the number of DEGs during the second recovery phase was 399, exceeding the levels observed during both heatwave periods. This pattern indicates that transcriptional regulation in *T. peltata* was not confined to the thermal stress phase but was more extensively activated during post-stress recovery. Accumulating evidence from studies on single thermal stress events suggests that recovery represents an active phase of coral acclimation, involving sustained molecular reorganization rather than a passive return to homeostasis [42,48,49]. In *Acropora hyacinthus*, host transcriptional reprogramming persists even when symbiont physiological recovery remains incomplete [49]; moreover, Thomas and Palumbi [42] demonstrated that host transcriptomic profiles remained significantly altered for months after bleaching despite seawater temperature normalization and symbiont recovery, indicating that prolonged molecular adjustment after thermal disturbance is a common feature in coral recovery. However, transcriptomic responses to repeated thermal stress remain largely unexplored in corals. In other marine invertebrates, such as the pearl oyster *Pinctada maxima*, repeated heatwave exposure has been shown to overwhelm the initial metabolic and molecular defense capacity, leading to impaired physiological performance [50]. Drawing on this parallel, the elevated DEG abundance during the second recovery phase in *T. peltata* likely reflects an even greater regulatory demand—extensive transcriptional reorganization associated with repair, metabolic adjustment, and acclimation—as the cumulative costs of successive heat stress exceed the recovery capacity established after the first event. Consistent with this interpretation, the second recovery phase in our study was accompanied by substantial holobiont reorganization: previous observations showed that repeated marine heatwave exposure induced microbiome restructuring and persistent alterations in the coral–Symbiodiniaceae relationship, including enrichment of stress-tolerant bacterial taxa and incomplete recovery of zooxanthellae density [24]. These physiological and microbial adjustments likely imposed increased regulatory demands on the host, thereby driving the large number of DEGs required for metabolic regulation, nutrient balance, and the restoration of homeostasis during recovery.

### 4.3. Gene Signatures Associated with Thermal Resilience in T. peltata

Several genes and functional domains consistently associated with stress tolerance across experimental stages may represent molecular components of thermal resilience in *T. peltata*. Persistent up-regulation of *Hsp20* suggests an important role in maintaining protein homeostasis during repeated thermal stress. Similar evidence from *Montastraea cavernosa* indicates that coordinated regulation of host heat shock proteins contributes to reduced cellular damage during thermal exposure [51]. In addition, genes associated with calcium signaling (*EF-hand motifs*), extracellular matrix remodeling (thrombospondin domains), and redox regulation (*cytochrome P450*, *NAD-binding oxidoreductases*, and alternative oxidase) indicate coordinated regulation of cellular protection, mitochondrial function, and skeletal maintenance. Alternative oxidase, in particular, may contribute to oxidative homeostasis by reducing mitochondrial reactive oxygen species production through alternative electron transport pathways.

Beyond these individual gene categories, a more integrated view of the host recovery capacity emerges from the dynamic expression of the Salivary secretion pathway (KEGG: ko04970) [52,53,54]. As this pathway is defined primarily from mammalian physiology, its coral-associated components are limited. However, several components of this pathway, including Na^+^/K^+^-ATPase and Ca^2+^-ATPase, are shared with general epithelial ion transport processes that are important for calcification and cellular homeostasis in corals [55]. During the first recovery phase, genes in this pathway showed a transient regulatory response, suggesting an initial attempt to restore ionic equilibrium and repair cellular damage. However, following the second heat stress, the expression of these genes was profoundly altered, indicating that the cumulative stress overwhelmed the short-term repair capacity, thereby exacerbating the disruption of essential physiological functions.

Together, these molecular signatures—from persistent chaperone expression and redox regulation to the transient activation and subsequent failure of ion-homeostasis pathways—suggest that thermal resilience in *T. peltata* is supported by coordinated regulation of proteostasis, redox balance, metabolism, and tissue remodeling. However, the heightened transcriptional demands during the second recovery suggest that capacity for recovery may be eroded under recurrent stress, consistent with Brown et al. [41], who proposed that although some corals may become stress-hardened, many others may undergo sensitization during repeated heatwaves.

### 4.4. Comparison with Heat-Tolerant Red Sea Corals

The transcriptional response of *T. peltata* contrasts with the rapid transcriptomic recovery reported for heat-tolerant Red Sea corals. In *Stylophora pistillata* from the Gulf of Aqaba, >94% of host genes return to baseline expression during recovery at 32 °C, a response thought to underpin the exceptional thermal tolerance of these populations [43]. In the present study, *T. peltata* exhibited fewer DEGs during the second heatwave than the first, yet the largest DEG set was observed during the second recovery phase. This pattern indicates that the transcriptional response to repeated heatwaves in *T. peltata* involves a more prolonged or complex regulatory reorganization, rather than a rapid return to baseline.

This divergence likely reflects differences in thermal history and symbiont composition. Northern Red Sea corals inhabit a chronically warm environment and have been proposed as a thermal refuge [46], whereas *T. peltata* in Xuwen experiences a seasonally variable thermal regime [22,23]. Corals adapted to chronic warmth may rely predominantly on constitutive thermal tolerance, whereas populations from seasonally variable environments depend more heavily on inducible responses that can be overwhelmed under severe or repeated heat stress [47,55]. Even *S. pistillata* from the Red Sea shows minimal recovery and high mortality at 34.5 °C [44], indicating that thermal resilience is not unlimited. This supports the conclusion that recurrent heatwaves may progressively constrain the inducible response capacity, even in species that display evidence of acclimation [41].

Collectively, these findings suggest that the responses of *T. peltatai* to repeated heatwaves do not conform to a classical priming model, in which prior exposure attenuates the response to subsequent stress. Instead, most shared genes showed enhanced responses during the second heatwave and second recovery, indicating a reorganized rather than primed transcriptional state. This contrasts with chronically heat-exposed Red Sea species, emphasizing that coral thermal responses are species-specific and shaped by regional thermal history.

## 5. Limitations

This study has several limitations that should be acknowledged.

First, our experiments were conducted on a single coral species, *T. peltata*, and our transcriptomic analysis focused exclusively on the coral host, excluding the symbiont transcriptome. Given the substantial interspecific variation in thermal tolerance and symbiont composition among scleractinian corals, and the central role of symbiotic algae in coral thermal responses and bleaching, our findings may not be directly generalizable to other coral taxa. Host-only data cannot fully resolve the molecular mechanisms underlying the holobiont response to recurrent heatwaves. Integrating both host and symbiont transcriptomes across multiple coral species will be needed to provide a more complete picture of coral resilience under intensifying MHW regimes.

Second, the 5-day recovery period between heatwaves may not be sufficient for full physiological recovery. Because no standardized recovery interval exists for experimental MHW studies on corals, this duration was based on our previous work on this species and reflects the relatively short recovery windows that may occur under natural conditions. Longer recovery periods should be examined in future studies to determine the minimum time required for complete restoration.

Third, the RT-qPCR validation in this study has two methodological limitations. The primers used for target genes lacked experimentally determined amplification efficiencies, which may affect the accuracy of relative quantification. In addition, only a single reference gene was used for normalization, which may not fully account for variation in RNA quality, reverse transcription efficiency, or sample loading. It should be noted, however, that the RT-qPCR analysis in this study was intended to validate the expression trends observed in the transcriptomic data, rather than to serve as an independent quantitative expression assay. Therefore, the statistical and reporting requirements differ from those of a dedicated gene expression quantification study. The RT-qPCR results should be interpreted as supporting the transcriptomic trends rather than as absolute expression values. Future studies should include primer efficiency validation and multiple stable reference genes to improve the reliability of RT-qPCR-based expression estimates.

## 6. Conclusions

In conclusion, our study reveals that repeated marine heatwaves induce stage-specific transcriptional reprogramming in *T. peltata*. The initial response involved broad stress-defense activation and incomplete physiological restoration, whereas re-exposure triggered a reorganized transcriptional response with reduced physiological damage. Notably, the largest number of differentially expressed genes occurred during the second recovery phase, underscoring that recovery is an active, resource-demanding process involving cellular repair, metabolic reorganization, and holobiont adjustment, rather than a passive return to baseline. Together with prior physiological and microbiome evidence, including the incomplete recovery of zooxanthellae density, chlorophyll a content, and microbial communities, these findings suggest that thermal acclimatization in *T. peltata* is associated with coordinated regulation across successive heatwave–recovery cycles. This work advances our understanding of coral responses to intensifying thermal disturbances and highlights metabolic and ion-homeostasis pathways as potential indicators for evaluating coral resilience under future climate scenarios.

## Figures and Tables

**Table 1:** Primer sequences used for quantitative real-time PCR (qPCR). *β-actin* used as internal reference.

Gene	Description	Primer Sequence (5′–3′)	Annealing Temperatures	Product Size
*β-Actin*	Reference gene	F: ATGACAGAGGATGAGGGTAGAAA R: GAGAAGAGGTCCCGACGAATA	57 °C	182 bp
*HSP20*	Protein processing in endoplasmic reticulum	F: TCAAATCCACCAAGCGGGAA R: CAGCGTACGTATCCTGTCCC	57 °C	165 bp
*EF-hand*	Calcium ion binding	F: CGGCTTTCTACATTGCAGGC R: GCGATTGACGGTTAACGAGC	57 °C	227 bp
*Thrombospondin type 1 domain*	Immune regulation	F: CGGTAGCTTCCTTGGGTTGT R: TTTGCACGACTGGATGGTGA	57 °C	192 bp
*H-type lectin domain*	Energy metabolism	F: TTGGCTGGAGGACATCACAC R: AAAAGAGGCTTGTGCAGGGA	57 °C	215 bp
*Glutathione S-transferase*, *C-terminal domain*	Oxidative stress	F: ATCAGCCACTGCTTCGTCAA R: ATGGGCCAAGGAGAAAGCAT	57 °C	191 bp
*Glutathione S-transferase*, *N-terminal domain*	Oxidative stress	F: GCCTTGTACTTGCCGCAAAA R: ACTCTGCCATCGGGATGTTC	57 °C	223 bp
*Oxidoreductase NAD-binding domain*	Oxidative stress	F: TTCTCGCTTCGCTTCCCTAC R: CTTGGTGCGGTGGACTTTTG	57 °C	214 bp
*Cytochrome P450*	Oxidative stress	F: TATAGGCGACCAACCATCGC R: GCCAAAGTCACGCAGTTGTT	57 °C	173 bp

**Table 2:** Summary of de novo transcriptome assembly results.

Length Range	Transcript	Unigene
200–300	45,005 (35.59%)	37,004 (37.01%)
300–500	48,626 (38.45%)	39,312 (39.32%)
500–1000	14,036 (11.10%)	10,090 (10.09%)
1000–2000	10,630 (8.41%)	7671 (7.67%)
2000+	8149 (6.44%)	5893 (5.89%)
Total Number	126,451	99,972
Total Length	82,919,313	62,541,149
N50 Length	1133	1029
Mean Length	655.74	625.59

## Data Availability

The sequencing data generated in this study have been deposited in the NCBI database under BioProject accession number PRJNA1050673. Other data supporting the findings of this study are available in the article.

## Supplementary Materials

The following supporting information can be downloaded at: https://www.mdpi.com/article/10.3390/biology15211929/s1, Table S1: Summary of transcriptome sequencing data quality assessment; Table S2: All Database annotation; Table S3: Supplementary of KEGG enrichment analysis of co-expression trends across the four experimental stages; Table S4: Supplementary for GO enrichment of all DEGs; Table S5: Supplementary for KEGG enrichment of all DEGs; Table S6: The expression of DEGs in the salivary secretion pathway at different stress and recovery stages in *T. peltata*.
